# Segmental duplication-quantitative fluorescent-polymerase chain reaction: An approach for the diagnosis of Down syndrome in India

**DOI:** 10.4274/tjod.56244

**Published:** 2018-03-29

**Authors:** Ambreen Asim, Sarita Agarwal

**Affiliations:** 1 Sanjay Gandhi Postgraduate Institute of Medical Sciences, Department of Medical Genetics, Lucknow, India

**Keywords:** Segmental duplication-quantitative fluorescent-polymerase chain reaction, aneuploidies, trisomy 21

## Abstract

**Objective:**

Early detection of high-risk pregnancies for Down syndrome (DS) is the main target of offering prenatal diagnosis. Segmental duplication-quantitative fluorescent-polymerase chain reaction (SD-QF-PCR) can be used as an alternative method for prenatal diagnosis of DS. SD-QF-PCR involves SD sequences between the test and control chromosomes to detect aneuploidies. SD are two similar sequences with different fragment lengths, located on two different chromosomes. When these SD regions are amplified, the peak ratio between the two different chromosomes remains as 0.9 to 1.1 and the trisomy 21 results in the ratio of 1.4 to 1.6.

**Materials and Methods:**

In this study, we applied SD-QF-PCR to detect the presence of trisomy 21 in 60 age-matched controls and 60 DS samples. The PCR amplification of SD regions is performed using a single pair of fluorescent-labelled primers, the peak ratio between the two different chromosome regions are evaluated.

**Results:**

All sixty control samples showed the peaks to range from 0.9 to 1.1, which was suggestive of normal samples, and peaks of 65 DS samples ranged from 1.4 to 1.6, which suggested the presence of trisomy 21.

**Conclusion:**

Segmental duplication quantitative fluorescent PCR is a sensitive and rapid aneuploidy detection technique and hence can be used as a standalone test to detect trisomy 21 as well as other aneuploidies.

## Introduction

Trisomy 21 is the main cause of Down syndrome (DS) and it is associated with various other clinical phenotypes such as Alzheimer’s disease, congenital heart diseases, cancers, Hirschsprung’s disease, leukemias, epilepsy, sleep disorder, infertility-related issues, and a various nutrient deficiencies. The incidence of trisomy 21 is 1 in 1000 live births; however, it differs among ethnic groups^(1)^. According to National Down Syndrome Society survey, the life expectancy for individuals with DS is 55 years^(2,3,4)^. DS is associated with various characteristic facial features such as hypotonia, craniofacial abnormality, flat facial profile, excessive skin at the nape of neck, hypotonia, hyper flexibility of the joints, dysplasia of the pelvis, anomalous ears, dysplasia of the mid phalanx of fifth finger, and a transverse palmer crease (simian crease) in early infancy^(4,5)^. Besides these, the other common features include an upward slant to the eye, flat nasal bridge, short neck, abnormally shaped ears, and white spots on the iris of the eye (called Brushfield spots)^(6)^. Most patients have mild-to-moderate intellectual disability. DS children can be prevented by offering a prenatal diagnosis to high-risk pregnancies. However, the sampling methods, chorionic villus sampling and amniocentesis are associated with a 0.5-1% risk of miscarriage^(7)^. Soft markers such as small or absent nasal bone, increased thickness of the nuchal fold, and the presence of large ventricles are used to detect the risk of trisomy in ultrasound at 12 to 24 weeks of gestation^(6,8)^. Cytogenic analysis is widely used as the gold standard method for offering a prenatal diagnosis. However, rapid aneuploidy testing methods such as fluorescent in situ hybridization (FISH), quantitative fluorescence-polymerase chain reaction (QF-PCR), and multiplex probe ligation assay (MLPA) are also routinely used for prenatal diagnosis in the laboratory^(4)^. A novel technique, segmental duplication-QF-PCR (SD-QF-PCR) was established by Kong et al.^(9)^ which involves SD sequences between test and control chromosomes to detect aneuploidies. SDs are two similar sequences with different fragment lengths, located on two different chromosomes. The method involves amplifying SD regions. When these sequences are amplified using a single pair of fluorescent-labelled primers, the peak ratio between the two different chromosomes remains as 0.9 to 1.1, and trisomy 21 results in the ratio of 1.4 to 1.6^(9,10)^.

## Materials and Methods

**Figure 1:**
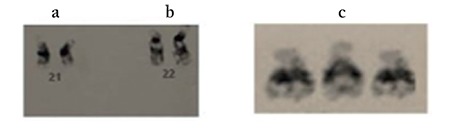
Chromosome as visualized on conventional karyotyping. a) Normal chromosome 21 pair, b) Normal chromosome 22 pair, c) Trisomy 21 showing presence of extra allele

The study included 60 patients with DS confirmed by karyotype (Figure 1) and 60 control samples after obtaining informed consent. Two milliliters of peripheral venous blood were collected in ethylenediaminetetraacetic acid from Sanjay Gandhi Postgraduate Institute of Medical Sciences, Lucknow, India. The study was approved by the institutional ethics committee (Sanjay Gandhi Postgraduate Institute of Medical Sciences, Lucknow, India) IEC code: 2014-140-PhD-79. The study was conducted in accordance with the code of Ethics of the World Medical Association (Declaration of Helsinki, 1975 revised in 2000) for experiments in humans. Genomic DNA isolation was performed using the standard phenol-chloroform method followed by PCR amplification using primers obtained from elsewhere [Muthuswamy and Agorwal^(10)^]. The PCR conditions included initial denaturation at 95 °C for 5 minutes, followed by 35 cycles of 30 seconds at 95 °C, 30 seconds at 60 °C, and 30 seconds at 72 °C, and a final extension step at 72 °C for 10 minutes^(9,10)^. Amplified PCR products (2 µL) were denatured with 8 µL HiDI and 0.5 µL LIZ at 95 °C for 5 minutes and loaded onto the genetic analyzer (ABI 310 Genetic Analyzer, Applied Biosystems). On the basis of the area acquired by the peak, the relative peak signal ratios were calculated. The expected value for a normal and trisomic sample are 0.9 to 1.1 and 1.4 to 1.6, respectively.

### Statistical Analysis

Statistical analysis was not required.

## Results

**Figure 2:**
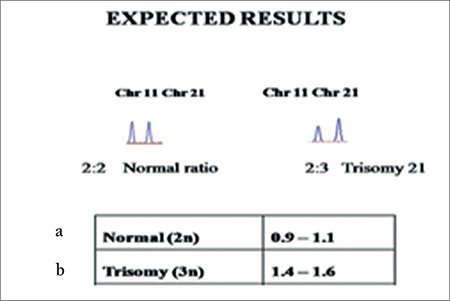
a) The ratios of the resulting peak obtained after normal or Down syndrome individuals, b) Ratios for normal and trisomy

**Figure 3:**
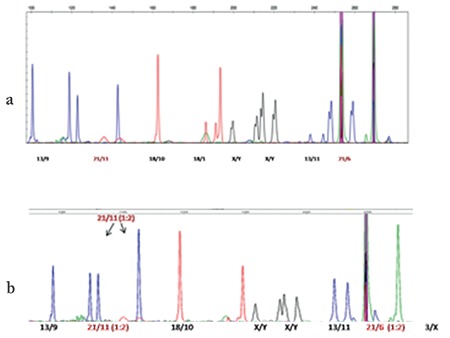
Results of segmental duplication-quantitative fluorescent-polymerase chain reaction. a) Normal individuals showing all normal sized alleles, b) Patients with Down syndrome showing 1:2 peak ratio for markers 21/11 and 21/6, respectively

SD-QF-PCR confirmed all 60 patients with DS to be positive for trisomy 21. Figure 2a shows the resulting peak in the case of euploids, the expected value was between 0.9 to 1.1, whereas for the trisomy, the value changes to 1.4 to 1.6, confirming the presence of an extra region. Figure 2b shows the expected value of the ratio for euploid, monosomy, and trisomy samples. Figure 3a shows the electropherogram obtained after SD-QF-PCR for euploid samples showing a normal allele ratio for both the markers, 21/11 and 21/6. Figure 3b shows the electropherogram obtained after SD-QF-PCR for trisomy patient samples showing values between 1.4 to 1.6 for markers 21/11 and 21/6, respectively, confirming the presence of DS.

## Discussion

The study aimed to confirm the use of SD-QF-PCR as an alternative method for postnatal diagnosis of DS, as well being usable for prenatal diagnosis. We recruited 60 age-matched controls and 60 DS samples and checked these samples for the presence of trisomy through the amplification of SD regions using a single pair of fluorescent-labelled primers. The peak ratio between the two different chromosome regions were evaluated. For euploid samples, the expected value was found to be between 0.9 and 1.1, and the expected value for trisomy 21 cases was found to be between 1.4 and 1.6. All samples were correctly diagnosed using the SD-QF-PCR method and the accuracy of the markers was found as 100%. SD-QF-PCR offers various advantages over other molecular based methods for both prenatal and post natal diagnosis of DS. Table 1 shows the list of all the techniques used for the diagnosis of Down syndrome. Cytogenetic analysis of metaphase chromosomes is performed on metaphase-stage fetal cells on amniotes creating unique banding patterns on the chromosomes. However, cytogenetic analysis is a time-consuming method and labor intensive^(4,9,10,11)^. MLPA can also be employed to evaluate the copy number of DNA sequences and offers a number of advantages such as simplicity of use, cost effectiveness, and requires a very short time for diagnosis. MLPA is divided into four steps: DNA denaturation, hybridization probe ligation, PCR amplification. After PCR amplification, the amplified products are loaded onto the genetic analyzer for capillary electrophoresis. The overnight hybridization step in this method makes MLPA labor intensive. However, MLPA is unable to detect low level mosaicism. The major drawback of MLPA is that it offers mosaicism or maternal cell contamination. MLPA also uses labeled probes, which are quite expensive, thus making this method costly^(12,13)^. The most widely used method for prenatal diagnosis is FISH, which is performed on interphase nuclei, using chromosome-specific fluorescent-labelled probes. The main drawback of FISH is that it is a low throughput method, involving hybridization of fluorescently-labelled chromosome-specific DNA^(4)^. In addition, sometimes diffuse signals are seen in the case of interphase chromosome^(14,15,16,17)^. An alternative method, QF-PCR is a short tandem repeat-based marker approach, which is present on chromosome 21, and by using these markers we can detect trisomy in 86.67% of cases with only two markers, and further, using a larger number of markers can increase the reliability of the test. Thus, QF-PCR is a robust, sensitive, and an automated technique that can handle many samples at a time. The main advantage of this techniques is that the diagnosis can be given within 12 hours^(4)^. Non-disjunction of parental origin can also be detected simultaneously. However, QF-PCR fails to detect mosaicism and various ploidy levels. However, these problems were overcome by the SD-QF-PCR method, in which various ploidy levels and maternal contamination can easily be detected. Thus, SD-QF-PCR is robust and cheaper than the above-mentioned methods. It is much faster approach than all the above-mentioned assays because the diagnosis can be given within 12 hours. The present report confers the use of SD-QF-PCR for rapid detection of aneuploidies for developing countries such as India. SD-QF-PCR can act as a standalone test for the detection of DS as well as other ploidy levels including monosomies. Furthermore, the present study, which was conducted in prenatal samples using genomic DNA from patients with DS, reported the sensitivity of this technique as 100%. Similar studies should also be conducted in prenatal samples from high-risk pregnancies for aneuploidies, which will further establish this technique as an alternative standalone test for the prenatal diagnosis of aneuploidies.

### Study Limitations

The present study was conducted in postnatal samples, however, SD-QF-PCR method should also be subjected in prenatal samples of DS as well. 

## Conclusion

The primary target of prenatal diagnosis is the early detection of high-risk pregnancies for DS. The choice after prenatal diagnosis of DS as to whether a pregnancy should continue a is a complex process because it involves various socio-economic factors. The risk for fetal trisomy can be evaluated on the basis of various factors such as prior family history, maternal age, fetal ultrasound makers, and biochemical tests of maternal serum. Women who are identified as high-risk carriers can receive genetic counseling and other additional tests such as cytogenic analysis and other molecular methods (FISH, QF-PCR, and MLPA) can be employed. However, these  methods have different disadvantages which were overcome by the novel SD-QF-PCR method. SD-QF-PCR is an automated, rapid, reliable, sensitive, and robust technique, and can be used for the diagnosis of various ploidy levels in a clinical setting.

## Figures and Tables

**Table 1:**
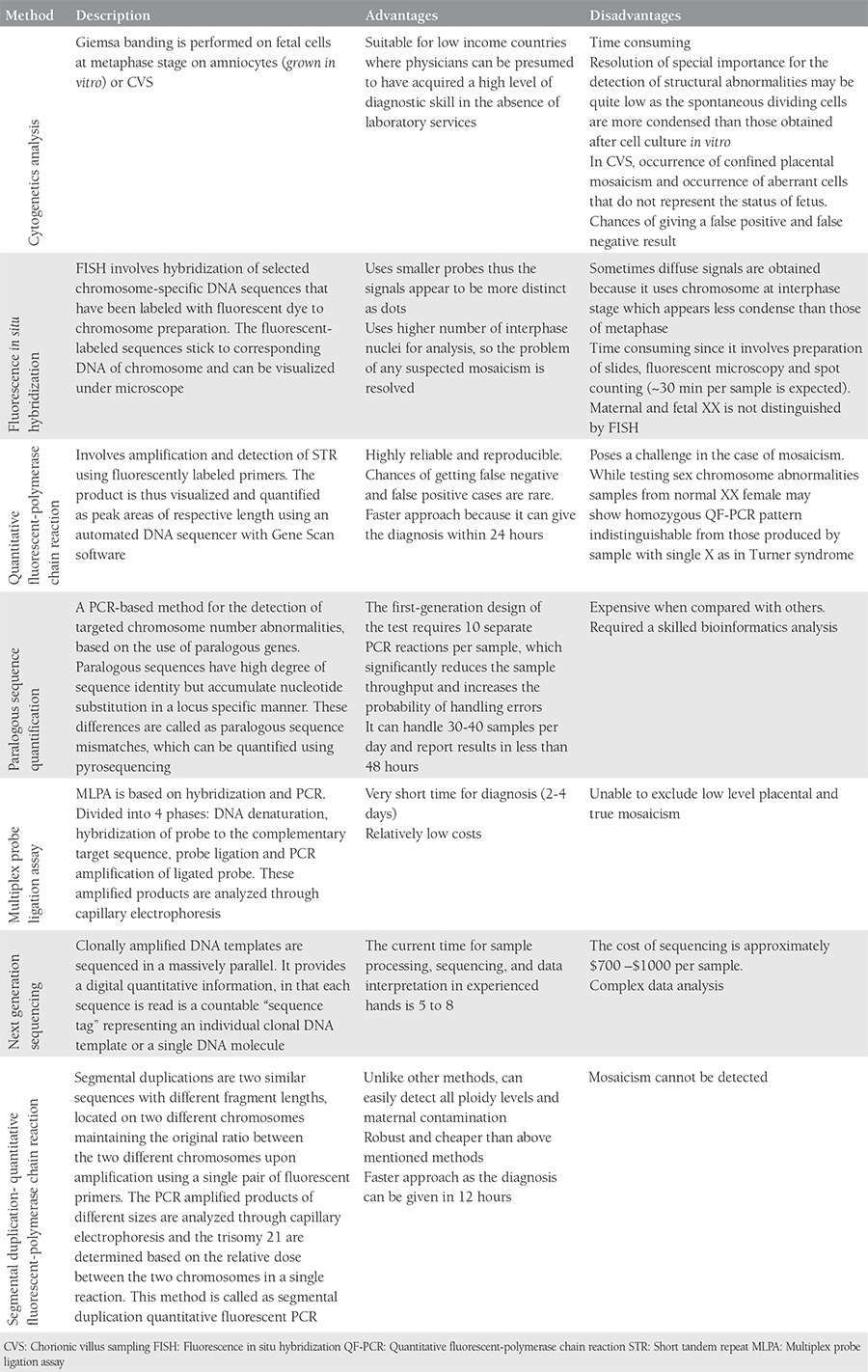
Description of techniques for diagnosis of Down syndrome Ambreen et al.^(4)^
